# Metadata analysis of retracted fake papers in Naunyn-Schmiedeberg’s Archives of Pharmacology

**DOI:** 10.1007/s00210-023-02850-6

**Published:** 2023-11-23

**Authors:** Jonathan Wittau, Roland Seifert

**Affiliations:** Institute of Pharmacology, Hannover Medical School, Carl-Neuberg-Straße 1, 30625 Hannover, Germany

**Keywords:** Fake paper, Paper mill, Naunyn-Schmiedeberg’s Archives of Pharmacology, Retracted, Metadata

## Abstract

An increasing fake paper problem is a cause for concern in the scientific community. These papers look scientific but contain manipulated data or are completely fictitious. So-called paper mills produce fake papers on a large scale and publish them in the name of people who buy authorship. The aim of this study was to learn more about the characteristics of fake papers at the metadata level. We also investigated whether some of these characteristics could be used to detect fake papers. For that purpose, we examined metadata of 12 fake papers that were retracted by Naunyn-Schmiedeberg’s Archives of Pharmacology (NSAP) in recent years. We also compared many of these metadata with those of a reference group of 733 articles published by NSAP. It turned out that in many characteristics the fake papers we examined did not differ substantially from the other articles. It was only noticeable that the fake papers came almost exclusively from a certain country, used non-institutional email addresses more often than average, and referenced dubious literature significantly more often. However, these three features are only of limited use in identifying fake papers. We were also able to show that fake papers not only contaminate the scientific record while they are unidentified but also continue to do so even after retraction. Our results indicate that fake papers are well made and resemble honest papers even at the metadata level. Because they contaminate the scientific record in the long term and this cannot be fully contained even by their retraction, it is particularly important to identify them before publication. Further research on the topic of fake papers is therefore urgently needed.

**Supplementary Information:**

The online version contains supplementary material available at 10.1007/s00210-023-02850-6.

## Introduction

Fake papers appear to be scientific papers but are fabricated and sold by professional forgers known as paper mills (Else and Van Noorden 2021). Reasons for publishing fake papers include pressure to publish, lack of time for research, and financial and professional benefits (Tian et al. 2016; Lin 2013; Quan et al. 2017). In the last few years, NSAP retracted 12 articles due to data fabrication and paper mill involvement (Seifert 2021). COPE & STM (2022) estimated the proportion of fake papers submitted to journals to be about 2%, but there are also much higher estimates (Sabel et al. 2023).

To preserve the integrity of the scientific record, it is essential to retract fake papers and prevent their further publication. Peer review alone does not seem to be sufficient for that purpose. Currently, there are various ways of detecting fake papers, all of which vary in effectiveness. Identifying fake papers manually, for example by searching for manipulated images (Byrne and Christopher 2020; van der Heyden 2021), is complex and requires intensive examination of the content of the papers. Often fake papers are discovered by chance (Seifert 2021) or by dedicated paper mill detectives (Else and Van Noorden 2021). There are also approaches to identify potential fake papers systematically on a larger scale. Scanning for papers with tortured phrases is one approach (Cabanac et al. 2021). Screening for papers with errors in nucleotide sequences is another way (Park et al. 2022). Identifying fake reviewers is a way to find many publications in one strike that were reviewed by the paper mills themselves (Day 2022). Scanning for extensively exchanged lists of authors between submissions of the same paper to different journals is a further approach (Wittau et al. 2023). Sabel et al. (2023) suggested that the use of non-institutional email addresses could be an indication of the presence of fake papers. Some approaches even use artificial intelligence to identify manipulated images (Van Noorden 2022). A recent study used machine learning to evaluate several features indicating paper mill work. The authors have created a decision aid to help people decide if they are looking at a fake paper (Dadkhah et al. 2023). An initiative called Integrity Hub is combining many different approaches in new tools that should help publishers to automatically detect fake papers in the future. These tools are said to use over 70 different features for identification. It is not transparent what these features are (Else 2022). Since all approaches have their limitations and paper mills will probably adapt and change practices over time, it is important to continue to study fake papers and look for as many ways as possible to detect them.

In this study, we examined metadata of retracted fake papers from NSAP to learn about their characteristics. This not only offers an interesting insight but is also important as metadata-based features could be useful for identifying fake papers. Therefore, this study also tested the usefulness of some metadata-based features that have already been proposed for this purpose. We also wanted to find out if some of the metadata were geographically biased, so using them could lead to discrimination against authors from specific regions. Finally, we took a look at whether the editorial board of the NSAP had discriminated against submissions from certain countries based on their experience with fake papers in the past.

## Methods

### Dataset

We collected metadata on all papers in the ‘original article’ category published by NSAP between 2017 and 2022, inclusive. The year 2017 was chosen as the beginning of this period, because the earliest fake paper detected in this journal in recent years was published in that year (status April 2023). We then separated the dataset into a group of proven fake papers and a group of supposedly honest publications. For the fake paper group, we used all publications that had been retracted by NSAP due to data manipulation, but where the authors had not initiated the retraction. Publications that had been retracted at the request of the authors were excluded from both groups, as we could not determine whether data errors were intentional or honest mistakes. The remaining papers in the dataset were then used as the reference group. Since another paper had been retracted in the meantime, the classification into fake papers and reference papers was updated to the current status on August 15, 2023. Our dataset contained the metadata listed in Table 1. Most but not all metadata were available for both groups. With one exception, which is noted in the corresponding figure, all metadata were collected in April 2023.

**Table 1:** Analyzed metadata

Metadata	Our source for this metadata
Publication and retraction dates	We got the reception date and the publication date from the official web version of each publication and calculated the publication time. Furthermore, we got the retraction date from the official web version of each publication. The time between publication and retraction was calculated too
Countries	We have taken the countries of origin of the authors' institutions as listed in the web version of the publications
ORCID IDs	We got the ORCID IDs from the web versions of the publications. We then evaluated whether at least one author per publication had provided an ORCID ID
Non-institutional email domains	We collected the domains of all email addresses of a publication that were given in the web version of the publication. The email domains were then automatically compared to three different lists of email domains. Two contained known academic email domains (https://pastebin.com/LND21t5F, accessed in May 2023; https://github.com/Hipo/university-domains-list, accessed in May 2023); the other one contained known commercial email domains (https://gist.github.com/ammarshah/f5c2624d767f91a7cbdc4e54db8dd0bf, accessed in May 2023). This allowed us to determine whether a publication had used non-institutional email addresses
Dubious references	We searched for retracted papers in the references by checking whether they were known to be retracted in PubMed (https://pubmed.ncbi.nlm.nih.gov/). Furthermore, we checked whether predatory journals were referenced by matching the journals of the referenced papers with the Beall’s list (https://beallslist.net/)
Performance metrics	We obtained the number of accesses to the publications from the web versions of the publications. Furthermore, we got the number of times a paper was cited from Web of Science (https://www.webofscience.com). We then calculated the mean value of accesses and citations per year to consider and work out that older publications had more time to be accessed and cited than newer ones
Citations before and after the retractions of the fake papers	We got the number of citations before and after the retractions from Web of Science (https://www.webofscience.com). We did not count citations that originated from retraction notes

### Conducted analyses

The next step was to evaluate all the metadata we had collected on the fake paper group. Whenever possible, the results were compared with those of the reference group. This allowed us to immediately assess whether the fake papers had unusual characteristics or were similar to the reference papers. This way, we were also able to test some features that had been suggested as fake paper indicators (Table 2).

**Table 2:** Features that have been proposed as identifiers for fake papers

Feature	Explanation	Literature
Short review times	There are reports of inappropriately short review times and faked reviews. A particularly short review time could therefore indicate peer review manipulation	Bishop 2023; Day 2022; Seifert 2021
Certain countries	Some countries were mentioned particularly often as the origin of fake papers	Else and Van Noorden 2021; Candal-Pedreira et al. 2022
No ORCID IDs	ORCID IDs allow the identification of authors, even if their first and last names are very common. The lack of an ORCID ID was mentioned as a possible characteristic of fake papers	Seifert 2021
Non-institutional email domains	The use of commercial respectively non-institutional email addresses was suggested as a feature of fake papers	Sabel et al. 2023; Seifert 2021
Dubious references	Fake papers may refer to other fake papers in order to generate citations for the paper mills' customers. Therefore, fake papers may be more likely to reference predatory journals or publications that have since been retracted	Beall 2012; Abalkina and Bishop 2022; Dadkhah et al. 2023

For some features that had been suggested as fake paper indicators, we tested whether there were regional differences in the reference group. The idea was to find out if using these features would discriminate against honest authors of certain countries.

We furthermore took a look if the fake paper cases had damaged the reputation of Chinese papers. For reasons of clarity, we have only compared China with other countries from which at least 5% of all published original articles in the period from 2017 to 2022, inclusive, originated.

### Data processing, statistics, and visualization

We used R 4.3.0 (R Core Team 2023) and R Studio (Posit team 2023) to process, analyze, and visualize the data. The R packages readxl (Wickham and Bryan 2023), dplyr (Wickham et al. 2023a, b), and tidyr (Wickham et al. 2023a, b) were used for data processing. To estimate the 95% confidence intervals for the mean differences between the fake paper group and the reference group, we used the Bootstrap Welch two-sample *t*-test. This was because the sample size of the fake paper group was small, not normally distributed, and the variances were not equal. The test was performed using the “boot.t.test” function from the R package MKinfer (Kohl 2023) with 9999 bootstrap replicates. We considered a mean difference to be statistically evident if the 95% CI did not include 0. The R packages ggplot2 (Wickham 2016), scales (Wickham and Seidel 2022), gridExtra (Auguie 2017), ggsignif (Ahlmann-Eltze and Patil 2021), maps (Becker et al. 2022), rgeos (Bivand and Rundel 2023), and rworldmap (South 2011) were used to create the plots.

## Results

In total, we collected metadata on 747 publications, of which 12 were proven fake papers (Table 3), 733 served as our honest reference papers and 2 were excluded from both groups.

**Table 3:** Proven fake papers

Paper no	Title	Bibliography
1	Antioxidant and antiapoptotic actions of selegiline protect against 3-NP-induced neurotoxicity in rats	Wahdan SA, Tadros MG, Khalifa AE (2017) Naunyn Schmiedebergs Arch Pharmacol. 390(9):905–917. https://doi.org/10.1007/s00210-017-1392-1
2	S-Adenosylmethionine synergistically enhances the antitumor effect of gemcitabine against pancreatic cancer through JAK2/STAT3 pathway	Liu Y, Bi T, Liu L, Gao Q, Shen G, Qin L (2019) Naunyn Schmiedebergs Arch Pharmacol. 392(5):615–622. https://doi.org/10.1007/s00210-019-01617-2
3	Treatment with a brain-selective prodrug of 17β-estradiol improves cognitive function in Alzheimer’s disease mice by regulating klf5-NF-κB pathway	Yan W, Wu J, Song B, Luo Q, Xu Y (2019) Naunyn Schmiedebergs Arch Pharmacol. 392(7):879–886. https://doi.org/10.1007/s00210-019-01639-w
4	Soyasapogenol B exhibits anti-growth and anti-metastatic activities in clear cell renal cell carcinoma	Wang L, Wang J, Zhao H, Jiang G, Feng X, Sui W, Liu H (2019) Naunyn Schmiedebergs Arch Pharmacol. 392(5):551–563. https://doi.org/10.1007/s00210-018-01607-w
5	Resibufogenin suppresses tumor growth and inhibits glycolysis in ovarian cancer by modulating PIM1	Li Q, Jiang C, Wang Y, Wei M, Zheng H, Xu Y, Xu X, Jia F, Liu K, Sun G, Zang J, Mo P (2019) Naunyn Schmiedebergs Arch Pharmacol. 392(12):1477–1489. https://doi.org/10.1007/s00210-019-01687-2
6	Fibrauretine reduces ischemia/reperfusion injury via RISK/eNOS activation	Wang C, Chang R, Gao G, Liu X, Zhang Y (2019) Naunyn Schmiedebergs Arch Pharmacol. 393(8):1515–1525. https://doi.org/10.1007/s00210-019-01770-8
7	Chrysophanol suppresses growth and metastasis of T cell acute lymphoblastic leukemia via miR-9/PD-L1 axis	Yin J, Yin Q, Liang B, Mi R, Ai H, Chen L, Wei X (2019) Naunyn Schmiedebergs Arch Pharmacol. 393(2):273–286. https://doi.org/10.1007/s00210-019-01778-0
8	Chrysophanol exhibits anti-cancer activities in lung cancer cell through regulating ROS/HIF-1a/VEGF signaling pathway	Zhang J, Wang Q, Wang Q, Guo P, Wang Y, Xing Y, Zhang M, Liu F, Zeng Q (2019) Naunyn Schmiedebergs Arch Pharmacol. 393(3):469–480. https://doi.org/10.1007/s00210-019-01746-8
9	Lupeol inhibits migration and invasion of colorectal cancer cells by suppressing RhoA-ROCK1 signaling pathway	Jiang Y, Hong D, Lou Z, Tu X, Jin L (2020) Naunyn Schmiedebergs Arch Pharmacol. 393(11):2185–2196. https://doi.org/10.1007/s00210-020-01815-3
10	Knock-down of LncRNA-XIST induced glioma cell death and inhibited tumorigenesis by regulating miR-137/SLC1A5 axis-mediated ROS production	Sun Y, Lv B, Zhang X (2020) Naunyn Schmiedebergs Arch Pharmacol. 394(3):557. https://doi.org/10.1007/s00210-020-01831-3
11	Physcion 8-O-β-glucopyranoside mediates the NLRP3-associated pyroptosis and cell metastasis in the human osteosarcoma cells via ER stress activation	Tian B, Hua Z, Wang Z, Wang J (2020) Naunyn Schmiedebergs Arch Pharmacol. 394(3):555. https://doi.org/10.1007/s00210-020-01836-y
12	LncRNA XIST inhibits ovarian cancer cell growth and metastasis via regulating miR-150-5p/PDCD4 signaling pathway	Wang S, Li G. (2020) Naunyn Schmiedebergs Arch Pharmacol. 394(4):763. https://doi.org/10.1007/s00210-020-01808-2

### Publication and retraction dates

**Fig. 1:**
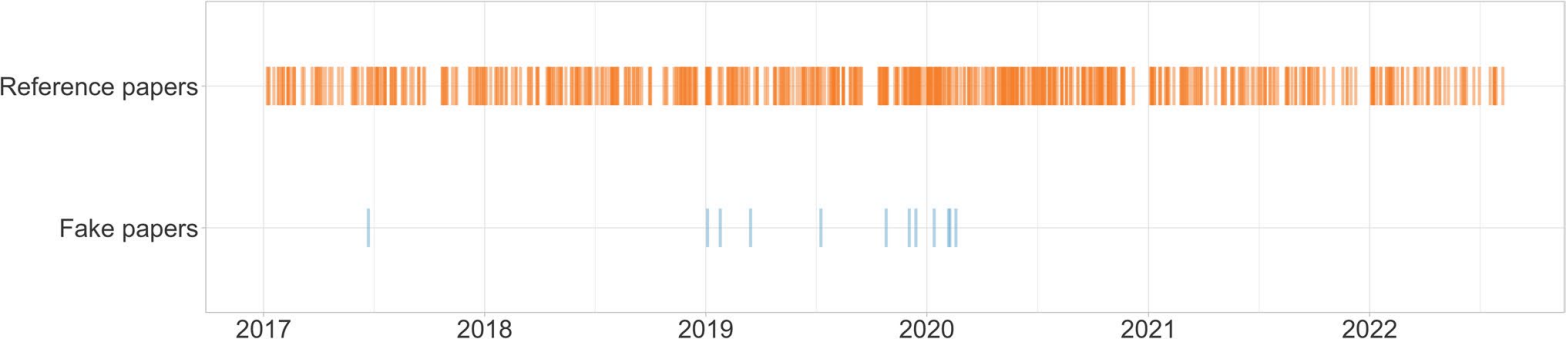
Publication dates

**Fig. 2:**
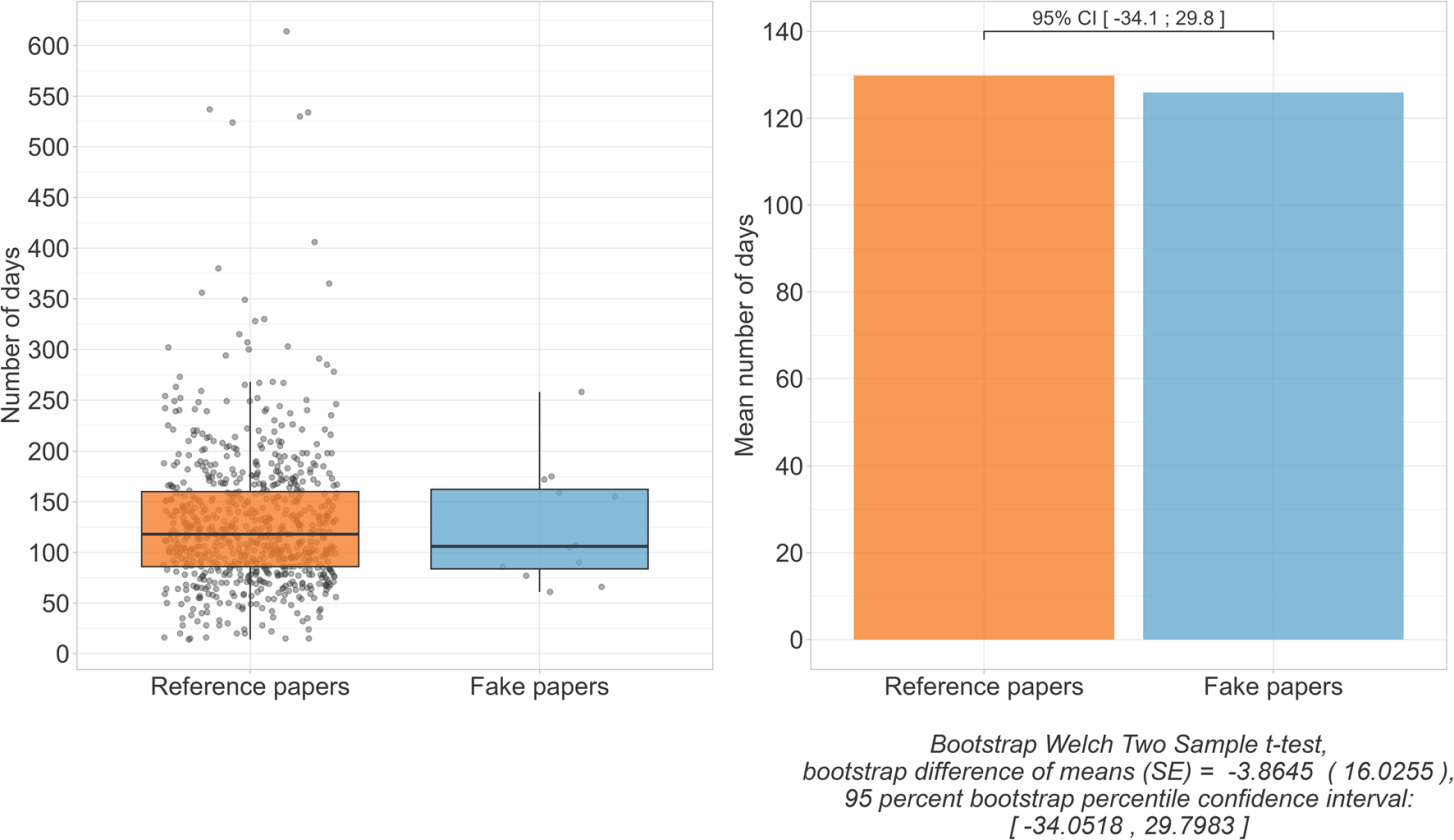
Time between submission and publication of a paper

**Fig. 3:**
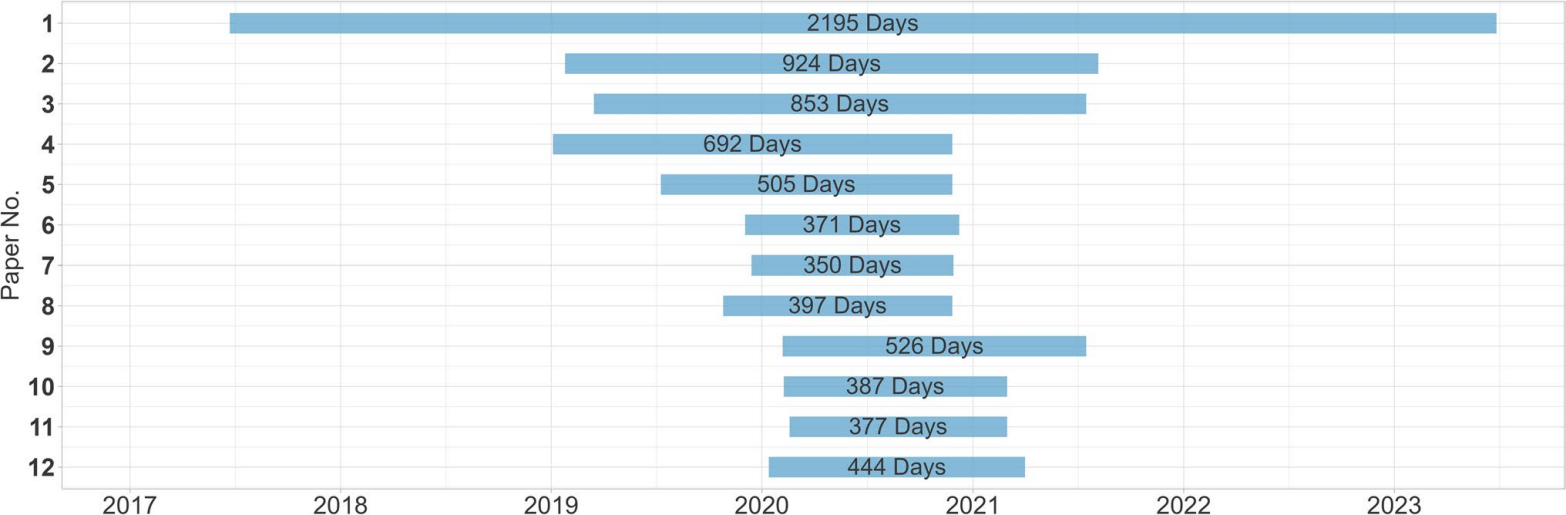
Time between publication and retraction of the fake papers**.** We got the retraction dates from the web versions of the publications. For papers **10**, **11**, and **12**, no retraction date was given, so we used the issue dates of the retraction notes

While there was also one fake paper published in 2017, all others appeared between early 2019 and early 2020. The reference papers were published at regular intervals between the beginning of 2017 and the end of 2022 (Fig. 1). With 125.9 compared to 129.8 days, the mean time from submission to publication of the fake papers was estimated to be 3.9 days shorter than for the reference papers, but the 95% CI [− 34.1;29.8] showed no statistical evidence for this. The publication time of the fake papers ranged from 61 to 258 days, while that of the reference papers ranged from 14 to 614 days (Fig. 2). Most of the fake papers were retracted between late 2020 and mid-2021, with one exception that was not retracted until 2023. Thus, the fake papers remained unrecognized in the scientific record for between 1 and 6 years (Fig. 3).

### Countries

**Fig. 4:**
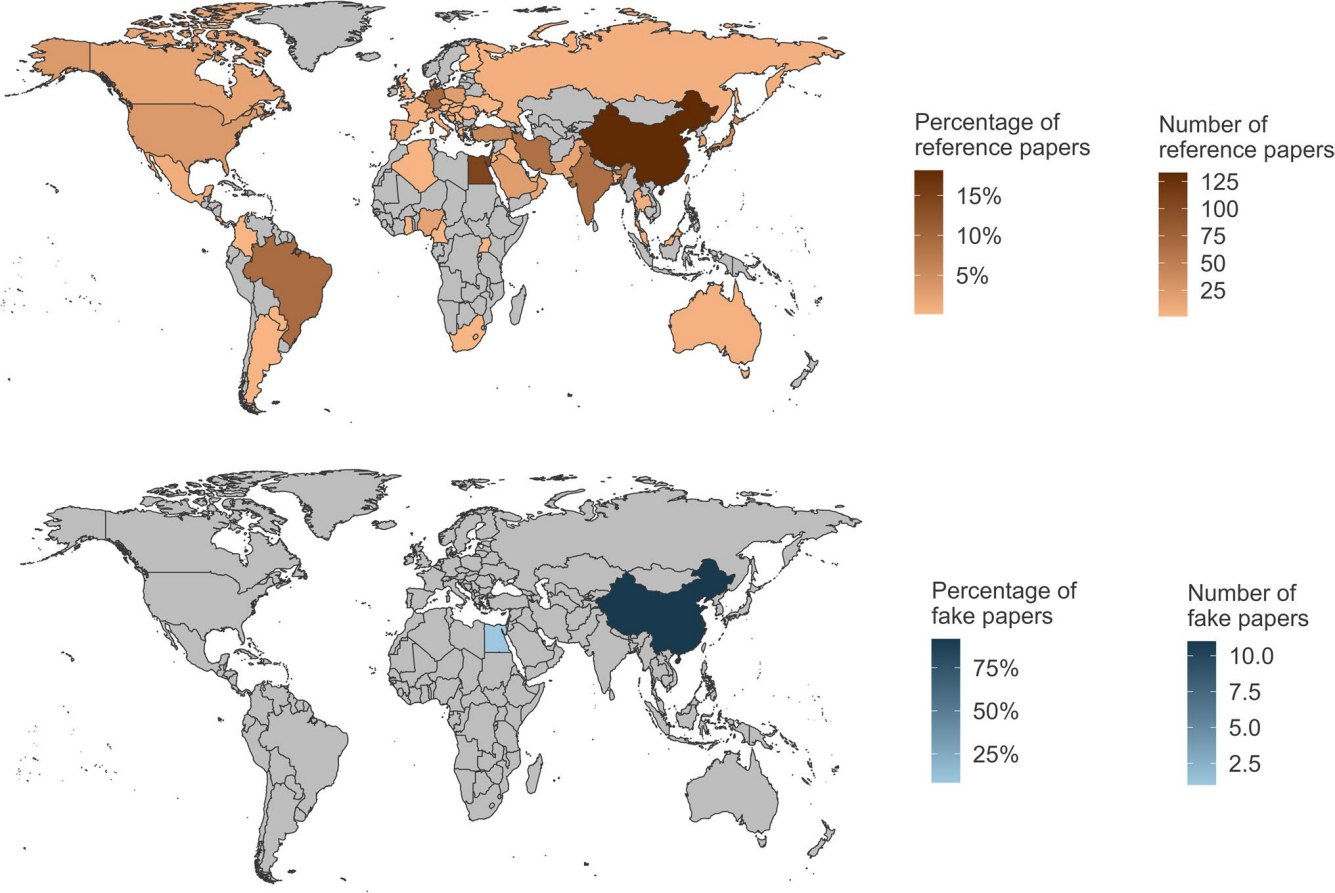
Origin of the fake papers and the reference papers**.** A publication may be counted more than once for different countries if it is the result of international cooperation

**Fig. 5:**
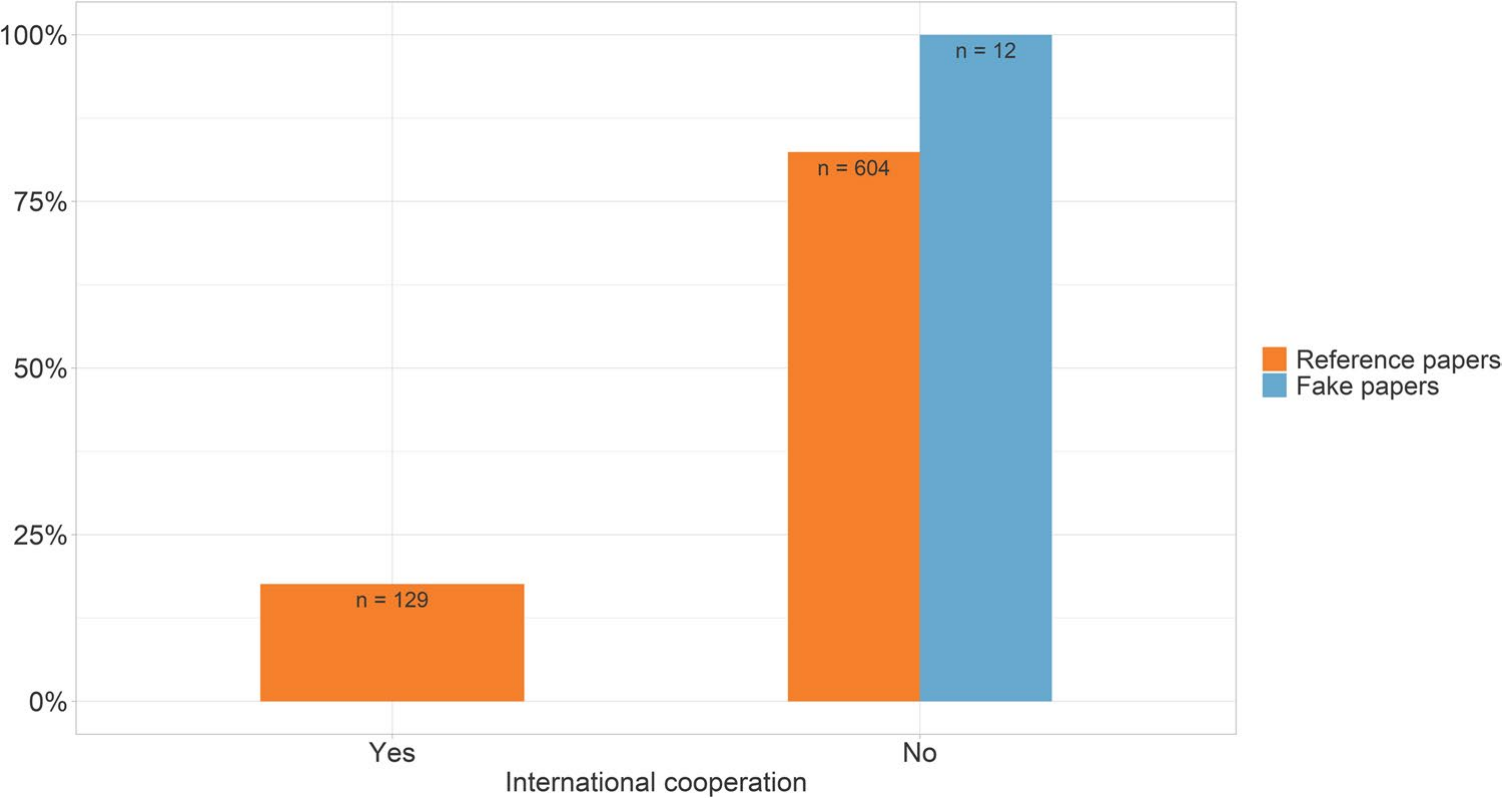
Proportion of publications with authors from different countries**.** If the institutions indicated by the authors on the paper are all from the same country, there is no international cooperation. If the authors are from institutions in different countries, this counts as international cooperation

Looking at the origin of the fake papers, it is striking that nearly all of them were from China (91.7%). Only one fake paper was from Egypt (8.3%) (Fig. 4). None of the fake papers had an international authorship (Fig. 5). In general, NSAP is an international journal with publications from many countries. However, most of the publications in the reference group were from China (18.1%), Egypt (14.6%), Germany (9.4%), Brazil (9.3%), India (8.9%), Iran (8.6%), Turkey (5.6%), and Japan (5.3%) (Fig. 4). 17.6% of the reference papers had authors from different countries (Fig. 5).

### ORCID IDs

**Fig. 6:**
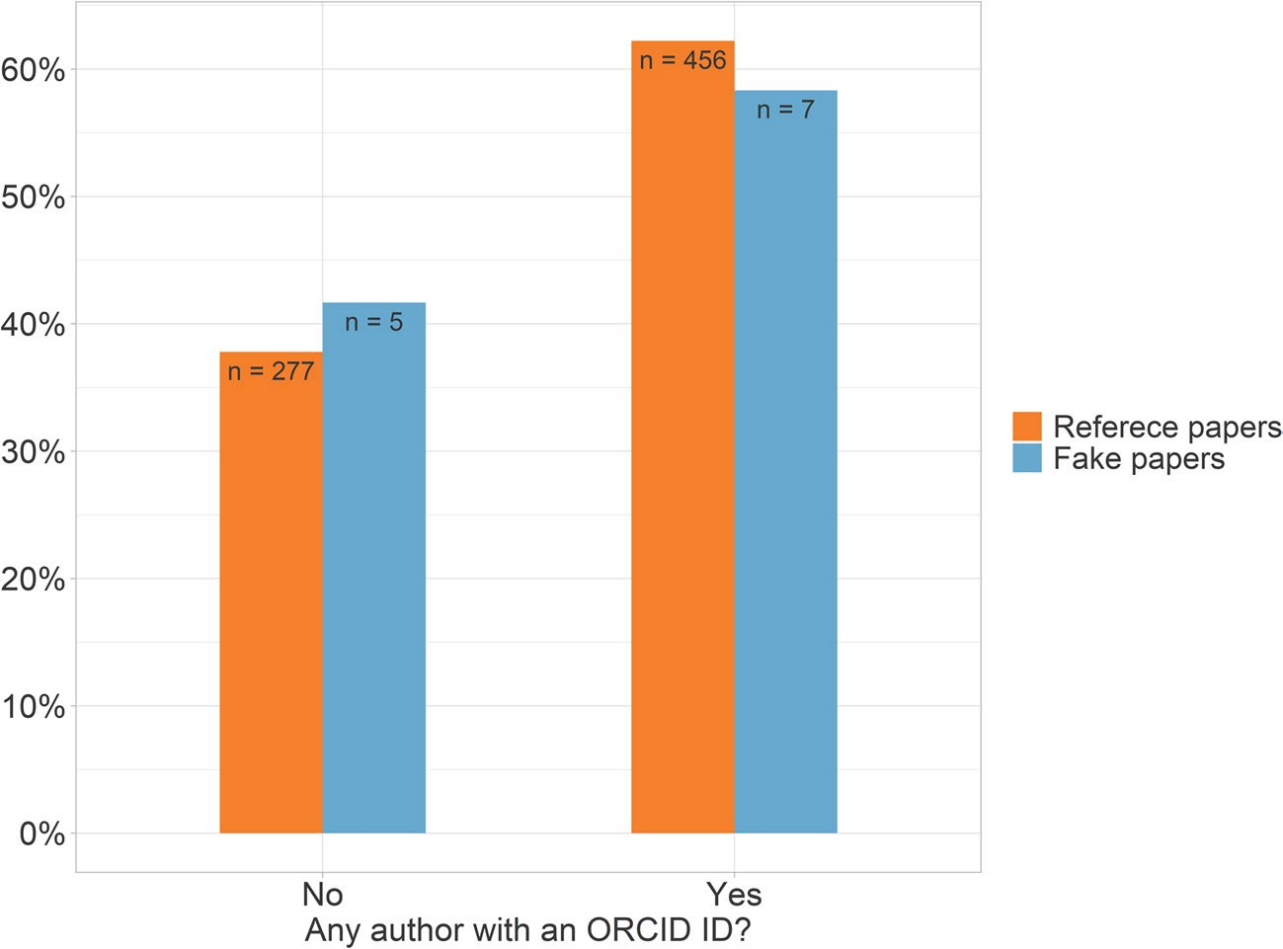
Proportion of publications with at least one author who provided an ORCID ID

**Fig. 7:**
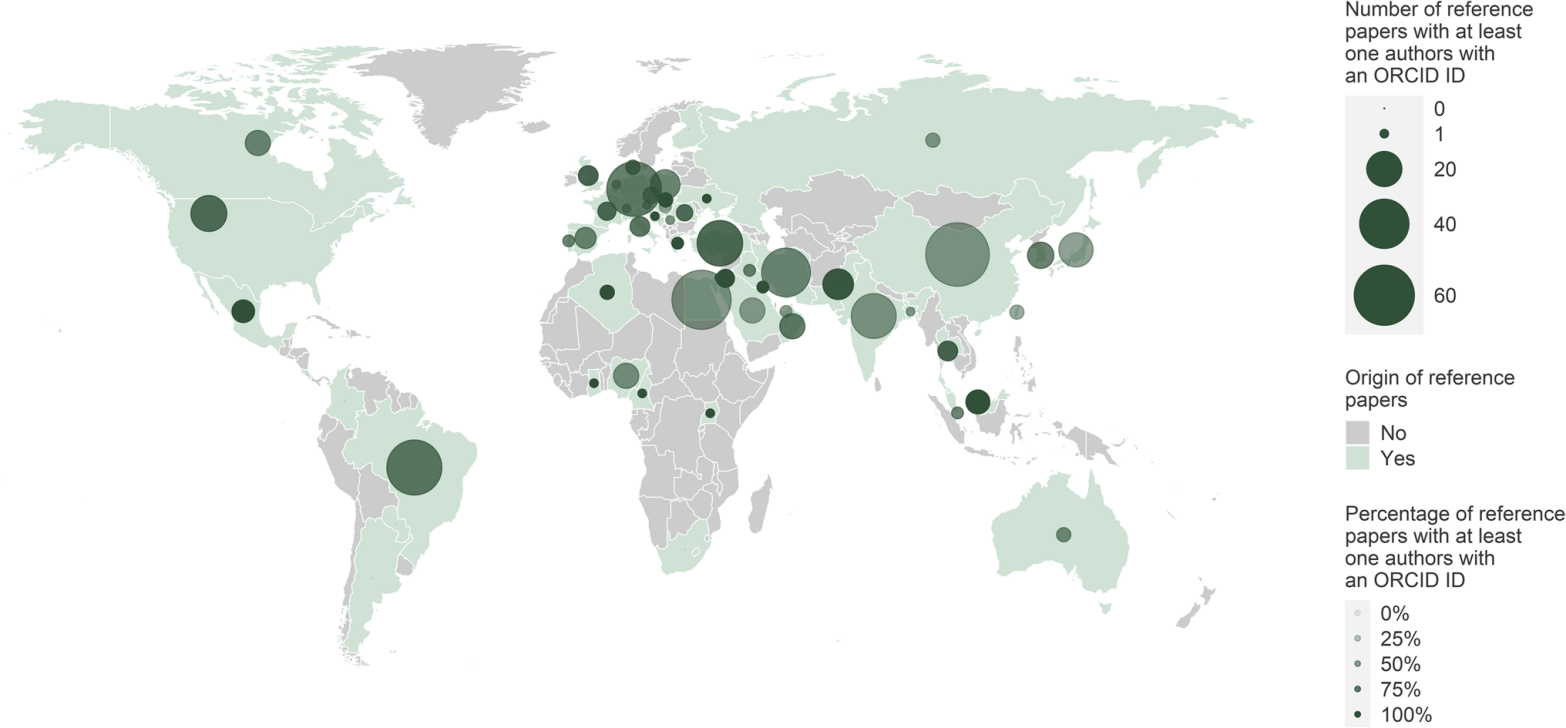
Regional differences in the use of ORCID IDs in the reference group**.** A publication may be counted more than once for different countries if it is the result of international cooperation

In percentage terms, fake papers were about as likely as reference papers to have at least one author who had provided an ORCID ID (58.3% respectively 62.2%) (Fig. 6). Instead, we found some differences in the likelihood that at least one of a paper's authors had provided an ORCID ID depending on the country from which a paper originated (Fig. 7).

### Non-institutional email domains

**Fig. 8:**
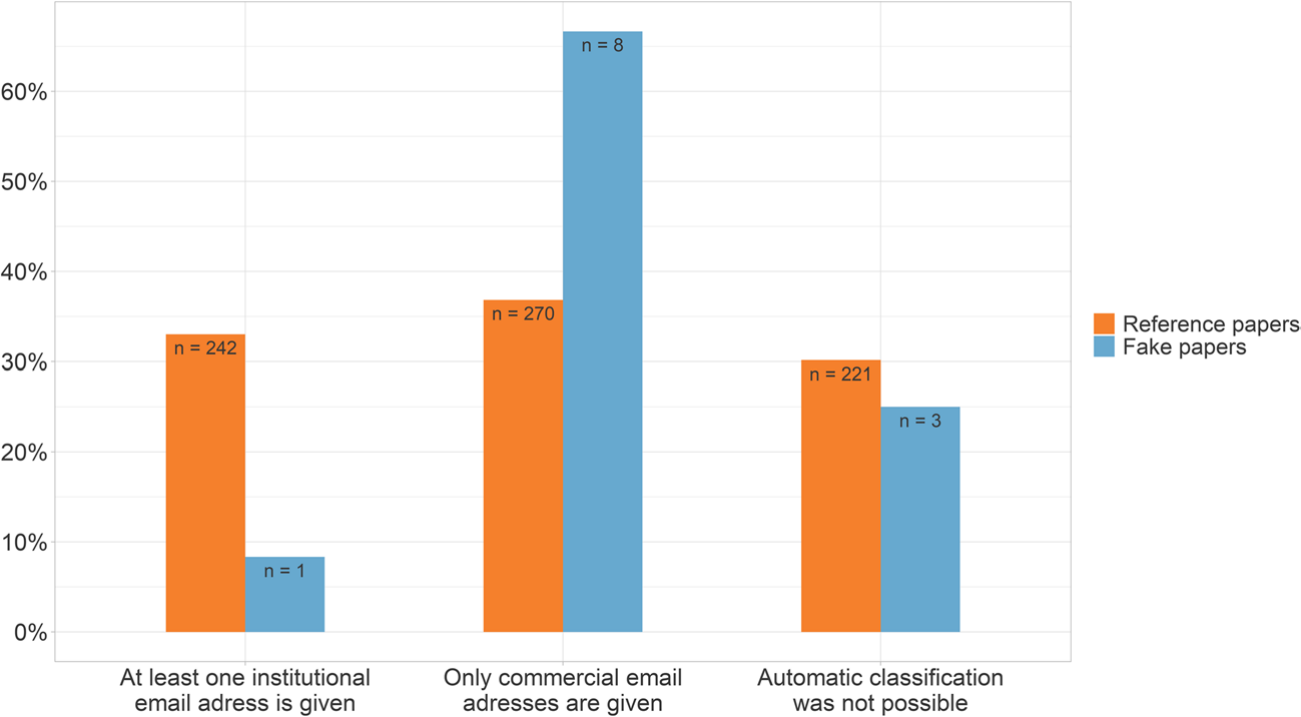
Proportion of publications with an institutional email address**.** In the cases where an automatic classification was not possible, either all given email domains were unknown, or a mix of unknown and commercial email domains was given

**Fig. 9:**
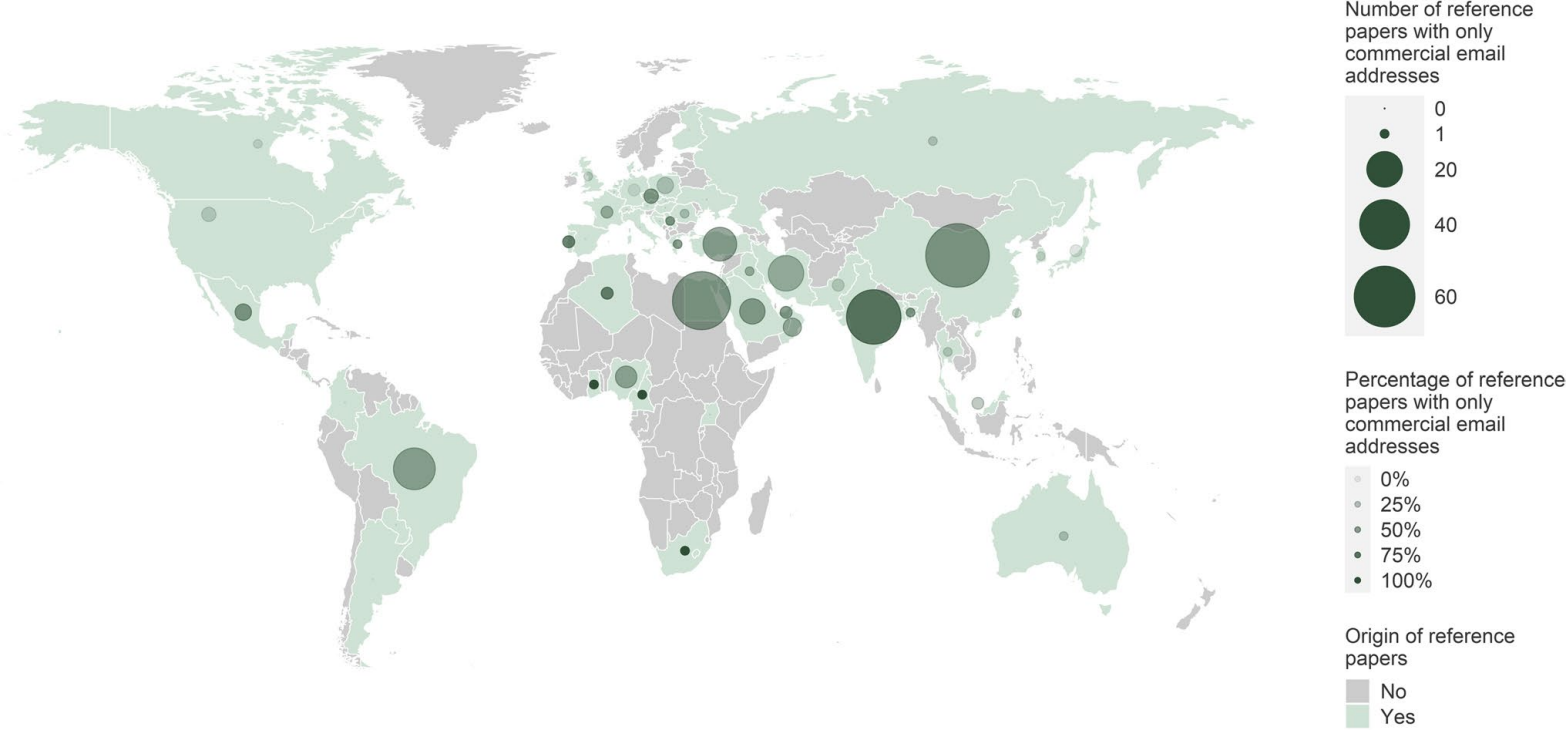
Commercial email addresses in the reference group by country**.** This map shows only those email domains that we could reliably identify as commercial (non-academic). However, the proportion of commercial email addresses may be higher than indicated on this map. A publication may be counted more than once for different countries if it is the result of international cooperation

At least 66.7% of the fake papers gave only a commercial email domain rather than that of an academic institution. In the reference papers, only email addresses with commercial domains were given in at least 36.8% of the cases. For both fake papers and reference papers, these numbers may be even higher, as in many cases we were unable to automatically classify email addresses as either commercial or institutional (Fig. 8). In the case of the fake papers, the commercial email domain 163.com was used five times, and 126.com was used three times. The five most popular commercial email domains in the reference group were gmail.com (87 papers), yahoo.com (67 papers), hotmail.com (37 papers), 163.com (34 papers), and 126.com (15 papers). Looking more closely at the countries of origin of papers from the reference group using commercial email addresses, it is striking that they are mainly from developing countries (Fig. 9). Particularly from China, Egypt, India, Brazil, Iran, Turkey, and Saudi Arabia, papers are regularly published in which only commercial email addresses are given.

### Dubious references

**Fig. 10:**
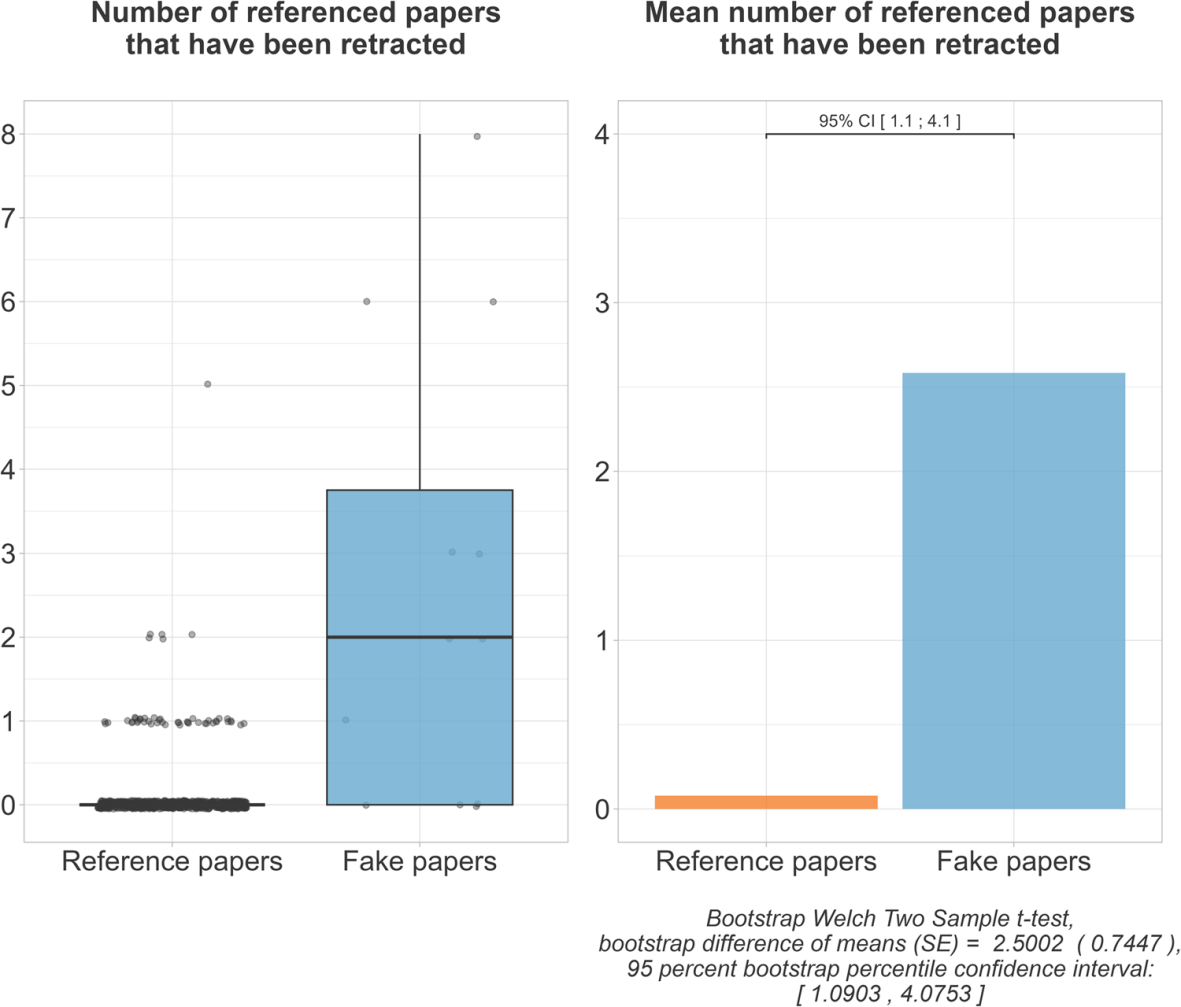
Referenced literature that has been retracted

**Fig. 11:**
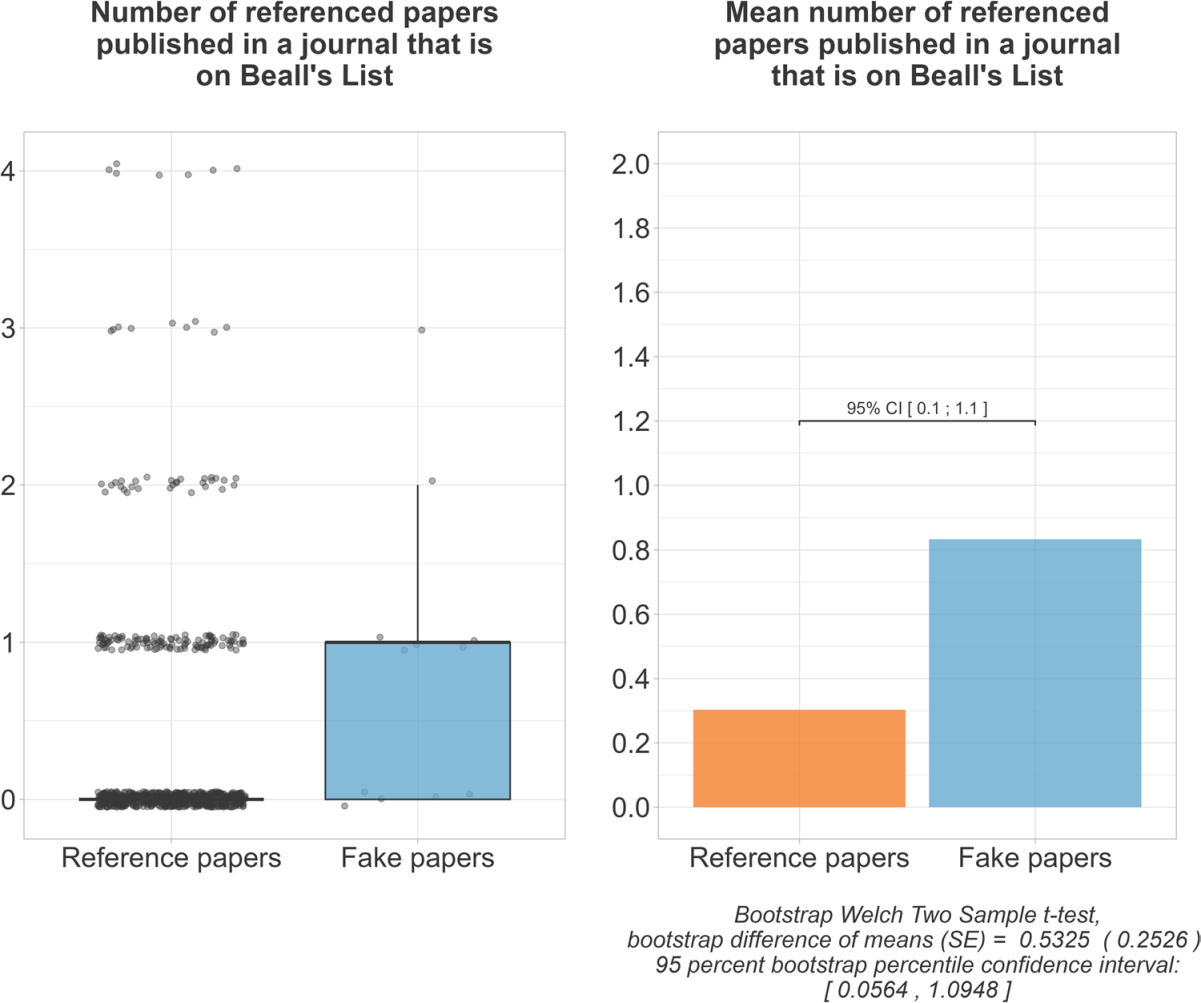
Referenced literature published in a journal that is on Beall’s List

Examination of the references showed that the fake papers more often referred to publications that had been retracted at the time of our investigation. The fake papers were estimated to reference 2.5, 95% CI [1.1;4.1] more retracted papers on mean. On mean, each fake paper referenced 2.6 retracted papers, while the papers in our reference group referenced on mean only 0.1 retracted paper each (Fig. 10). We further found that the fake papers were more likely to refer to papers published in a predatory journal. The fake papers were estimated to reference 0.5, 95% CI [0.1;1.1] more publications in predatory journals on mean. On mean, each fake and each reference paper referred to 0.8, respectively, 0.3 publications in predatory journals (Fig. 11).

### Performance metrics

**Fig. 12:**
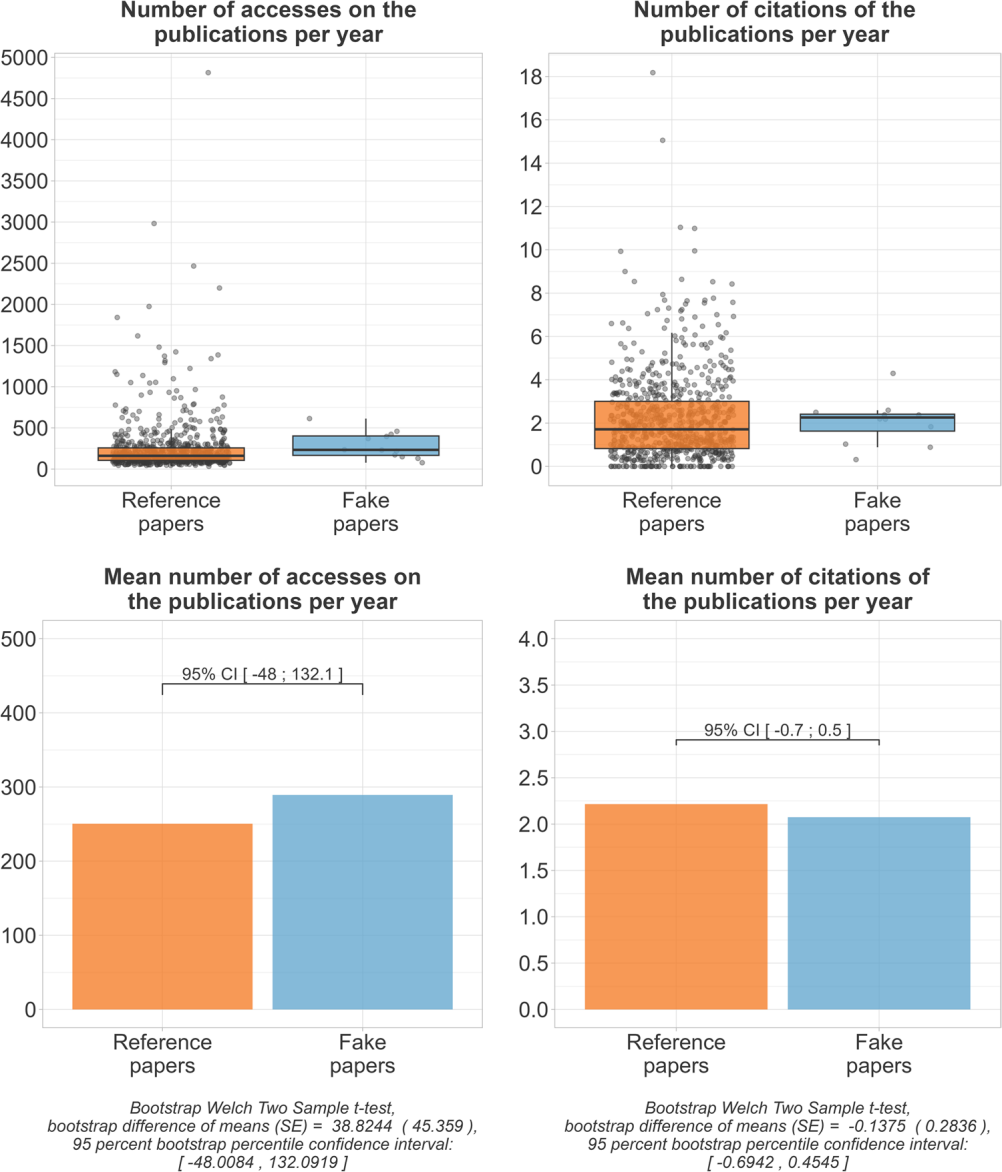
Performance metrics

Both groups performed similarly on the performance metrics. On mean, the fake papers were estimated to be accessed an additional 38.8, 95% CI [− 48;132.1] times per year. While, on mean, the fake papers got accessed 289.3 times per year, the reference papers got accessed only 250.5 times per year. The estimated difference in mean citations per year was  − 0.1, 95% CI [− 0.7;0.5]. The fake papers were cited an average of 2.1 times per year, while the reference papers were cited an average of 2.2 times per year (Fig. 12).

### Citations before and after the retractions of the fake papers

On mean, each fake paper was cited 4.3 times before being retracted. After the retraction, each paper was cited an average of 4.1 additional times. Thus, on average, 49.5% of the citations occurred after the retractions. The most cited fake paper was even cited exclusively after retraction (Table 4). Citations that came from retraction notes were not included in these numbers.

**Table 4:** Citations before and after the retraction of the fake papers

Paper no	Total citations	Citations before retraction	Citations after retraction	Percentage of citations after retraction
1	8	7	1	12.5%
2	10	8	2	20%
3	9	3	6	66.67%
4	7	1	6	85.71%
5	9	4	5	55.56%
6	2	1	1	50%
7	7	1	6	85.71%
8	14	0	14	100%
9	6	5	1	16.67%
10	6	3	3	50%
11	1	0	1	100%
12	5	2	3	60%

The number of citations is taken from Web of Science. Citations that originate from retraction notes have not been counted in this table. All citing papers published after the retraction of the respective fake paper count as citation after retraction. If a citing paper was published in the same month that the retraction occurred or before, it was considered a citation before retraction. The publication date of a citing paper is also taken from Web of Science. The data in this table were collected on October 02, 2023, later than the other data in this study. For this reason, the number of citations in this table differs in part from the number of citations used to calculate citations per year in Table 5 and Fig. 12

For a better overview, many results from above are summarized in Table 5.

**Table 5:** Overview of the results

Metadata		Results		Mean estimated difference, CI
		Reference group (733 papers)	Fake paper group (12 papers)	
Publication and retraction dates	Publication dates	Published at regular intervals between 2017 and 2022	91.7% (11) published between early 2019 and early 2020, 8.3% (1) Published in 2017	-
	Publication time	129.8 days average publication time	125.9 days average publication time	− 3.9, 95% CI [− 34.1;29.8]
	Retraction dates	-	91.7% (11) retracted between late 2020 and mid-2021, 8.3% (1) Retracted in 2023	-
	Time between publication and retraction	-	668.4 days on mean with a range of 1 to 6 years	-
Countries		18.1% (133) papers from China, 14.6% (107) papers from Egypt	91.7% (11) papers from China, 8.3% (1) papers from Egypt	-
ORCID IDs		62.2% (456) provided at least one ORCID ID	58.3% (7) provided at least one ORCID ID	-
Non-institutional email domains		36.8% (270) used only commercial email domains	66.7% (8) used only commercial email domains	-
Dubious references	Retracted references:	0.1 retracted paper was referenced on mean	2.6 retracted papers were referenced on mean	2.5, 95% CI [1.1;4.1]
	References in predatory journals:	0.3 predatory journals were referenced on mean	0.8 predatory journals were referenced on mean	0.5, 95% CI [0.1;1.1]
Performance metrics	Accesses per year	250.5 times per year accessed on average	289.3 times per year accessed on average	38.8, 95% CI [-48;132.1]
	Citations per year	2.2 times per year cited on average	2.1 times per year cited on average	-0.1, 95% CI [-0.7;0.5]
Citations before and after the retractions of the fake papers		-	Average of 4.3 citations before the retractions, average of 4.1 citations after the retractions	-

### Did the fake papers damage the reputation of Chinese papers in NSAP?

Since almost all fake papers retracted by NSAP were from China, we wanted to find out if the editorial board of NSAP had changed their treatment of Chinese publications now. This could have led to discrimination against honest Chinese authors. To find that out, we looked at the number of publications and the average publication time of those countries that publish the most papers in the NSAP. We also looked at the average number of times papers from these countries were cited to see if Chinese papers had lost reputation among NSAP readers. For the following analyses, we used all papers in our dataset that were from the countries with the most submissions to NSAP (see “Methods”), regardless of whether they were classified as fake papers or reference papers.

**Fig. 13:**
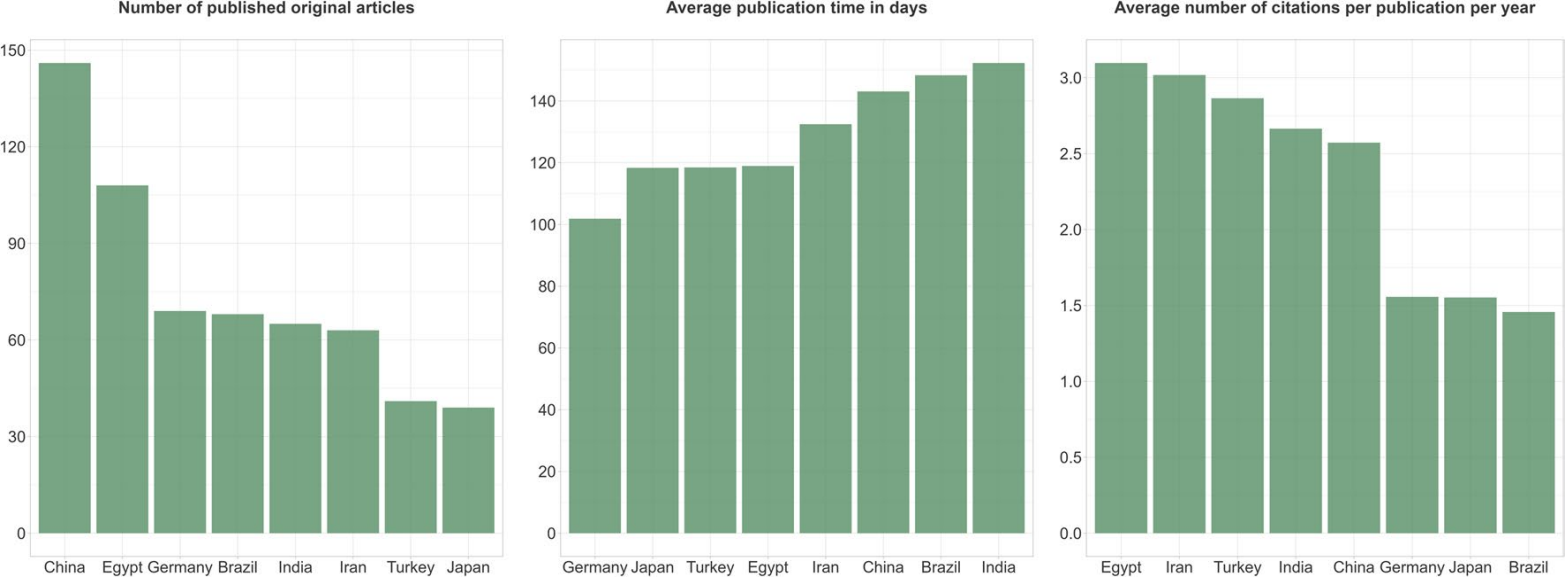
Publication metrics of the countries with the most submissions to NSAP for the period from 2017 to 2022 inclusive. All 747 publications from our dataset are included in this figure, regardless of whether they were classified as fake papers or reference papers. A publication may be counted more than once for different countries if it is the result of international cooperation

Looking at the whole period from 2017 to 2022 inclusive, with 146 publications, China is clearly the country from which most publications originate. On average, it takes about 143 days for Chinese papers to be published in NSAP. The average publication time for papers from the eight countries we compared here is about 129 days. This means that papers from China take about 14 days longer than average to be published in NSAP. Publications from China are cited on average 2.6 times per year, which is slightly above the average of 2.3 citations per year (Fig. 13).

**Fig. 14:**
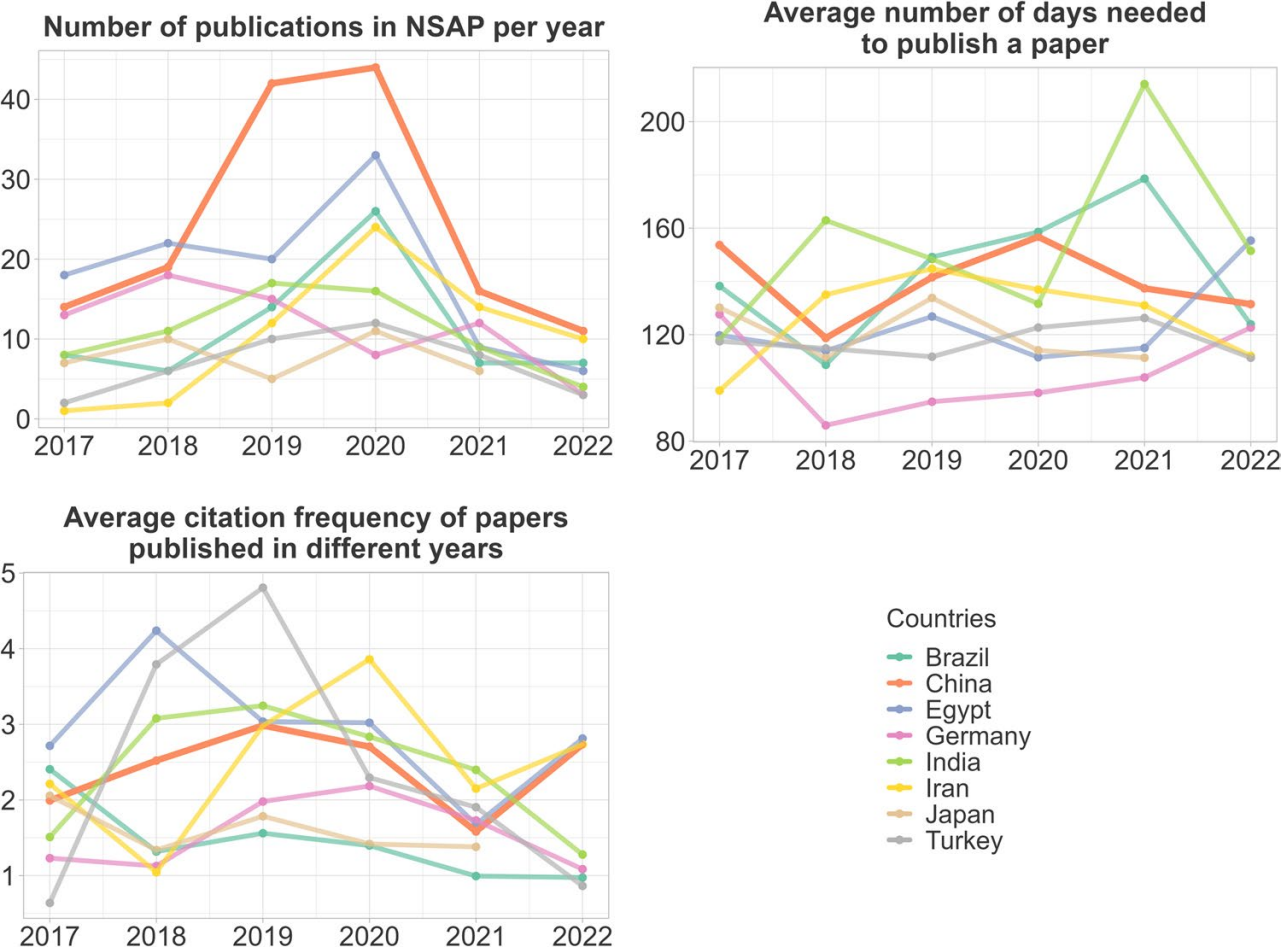
Development of the publication metrics of the countries with the most submissions to NSAP. All 747 publications from our dataset are included in this figure, regardless of whether they were classified as fake papers or reference papers. A publication may be counted more than once for different countries if it is the result of international cooperation

Most of the fake paper cases we investigated have been known to the NSAP since 2020, even if many of the retractions occurred later due to time-consuming procedures. For this reason, we have not only looked at the above data for the entire period, but also at its development over time. In 2021 and 2022, the number of publications from China as well as the publication time and the number of citations per publication per year for Chinese publications did not develop very different from the other six countries (Fig. 14).

## Discussion

We studied metadata of fake papers to learn about their characteristics. To assess the data, we compared them with a reference group of supposedly honest papers. This way, we also tested whether some features that had been suggested as potential indicators of fake papers were actually useful for this purpose (Table 6).

**Table 6:** Usefulness of proposed features for identifying fake papers

Feature		Evaluation of the proposed features for identifying fake papers
Short review times		Feature is not useful: There is no evidence of a mean difference between both groups
Certain countries		Feature is of limited use: Most fake papers are from China, but most Chinese papers are not fake. This feature discriminates against honest scientists from certain countries
No ORCID IDs		Feature is not useful: There is no relevant difference between both groups
Non-institutional email domains		Feature is of limited use: This feature is not specific. Fake papers are more likely to use commercial email domains, but many reference papers do as well. Geographical differences may lead to discrimination
Dubious references	Retracted references:	Feature is of limited use: In combination with other features, this feature might be a good indicator of fake papers
	References in predatory journals:	Feature is not useful: There is no relevant difference between both groups

### Publication and retraction dates

With one exception, all fake papers in our dataset were published between early 2019 and early 2020. That fake papers were published more frequently during this period does not seem unusual, as another study also reports that the number of published fake papers peaked in 2019 (Candal-Pedreira et al. 2022). In our case, a speculative explanation could be that all these fake papers came from a single paper mill that was attacking the NSAP during this period. This would also be supported by the fact that identical figures were found in some of the fake papers, as can be seen from the retraction notes. That no further fake papers were published after the beginning of 2020 could also be an indication that a single paper mill has ceased its activity at NSAP. However, it is also possible that fake papers were published before and after but have not yet been discovered. If it is indeed a common practice of paper mills to submit various fake papers to the same journal multiple times within a relatively short time span, all journals in which a fake paper has been discovered should search particularly careful for further fake papers around that time. COPE & STM (2022) already reported similar.

The time that the fake papers took from submission to publication was inconspicuous. We did not find statistical evidence for a difference between the mean publication times of both groups. Probably, the publication time of the fake papers was so average because the publication processes were completely unremarkable. This would underline that the fake papers are done quite professionally.

In recent publications, abnormally short review times were suggested as a sign of fake papers, as this may indicate manipulated peer review (Bishop 2023; Day 2022). Seifert (2021) even reported on reviews that were delivered in minutes. Dadkhah et al. (2023) did not find short review times to be a key feature of fake papers but rather an indicator of suspicious papers. We did not have data on the review times themselves but used the (online) publication times as an approximation. This period also includes the time needed for editorial work. As mentioned above, we did not find evidence of shorter publication times for the fake papers. The minimal publication time for a fake paper was about 2 months, which is not strikingly short. However, it is also possible that NSAP editors requested additional reviewers when reviews were delivered unusually fast and that short review times therefore cannot be read from the publication time.

For most of the fake papers, it took between one and three years until they were retracted. In one case, it even took six years. Sadly, this is a long time during which these papers were considered honest.

### Countries

Although the proportion of NSAP publications from China and Egypt was generally high, the geographical distribution of fake papers did not correspond to that of the reference papers. China is very overrepresented here (about 92% of the fakes but only about 18% of the reference papers are from China). Other studies also mention China as a major country of origin for fake papers. However, the fake paper problem is not limited to China, some other countries are mentioned too (Else and Van Noorden 2021; Candal-Pedreira et al. 2022). Dadkhah et al. (2023) recommend an additional inspection of a paper when it originates from certain countries. Even though fake papers seem to be more likely to originate from certain countries, we recommend being very careful about using this as a feature for fake paper identification. This could lead to discrimination against honest scientists from certain regions.

### ORCID IDs

ORCID IDs allow the identification of authors, even if their first and last names are very common. Seifert (2021) noted that few fake paper authors had provided ORCID IDs in their papers. Dadkhah et al. (2023) suggested missing ORCID IDs as a feature that could indicate fake papers. However, in our study, the fake papers were just as likely to have at least one author with an ORCID ID as the reference papers. Since we observed geographical differences in this feature, using it could lead to discrimination. However, we only checked whether any author of the paper had provided an ORCID ID, but not how many authors, and neither did we check the information in the ORCID profile.

### Non-institutional email domains

About two-thirds of the fake papers in our dataset gave only commercial email addresses. This was twice the rate of our reference group. There were also regional differences in the use of commercial email addresses. Most of the papers in our reference group that had exclusively stated commercial email addresses were from developing countries. Since all fake papers in our dataset were from developing countries as well, the high share of fake papers using commercial email addresses may be biased. That scientists from developing countries use commercial email addresses more often has been observed before (Shen et al. 2018). Possible reasons could be that institutional email addresses in these countries are sometimes less reliable and functional than those of large international email providers (Rousseau 2018).

In a recent study, the exclusive use of commercial (non-academic) email addresses in publications was presented as a good feature for identifying fake papers (Sabel et al. 2023). Another study found that commercial email addresses can make a paper suspicious (Dadkhah et al. 2023). Based on our data, we found this feature to be of limited use. For one thing, more than a third of all honest papers in our data set also used exclusively commercial email addresses, so scanning for this feature would lead to many false positives. For another, using commercial email addresses as a feature to identify potential fake papers could lead to discrimination against honest scientists from developing countries. Furthermore, even in developed countries, there are good reasons for authors to use commercial rather than academic email addresses. For example, many authors in this forum (https://www.researchgate.net/post/Is_it_acceptable_to_use_a_gmail_address_as_contact_information_for_a_corresponding_author_on_a_publication) point out that commercial email addresses remain permanently valid and do not change when the institution is changed.

### Dubious references

We found that the fake papers were more likely to refer to dubious literature. The evidence for a more frequent referencing of predatory journals, on mean, was weak, and we did not consider it meaningful. More relevant was that, on mean, the fake papers referred to more retracted literature. It is imaginable that these retracted references were other fake papers that, before being exposed, were referenced to generate citations for other publications from the same paper mill. Abalkina and Bishop (2022) had similar thoughts when they found inappropriate references that had nothing to do with the context in fake papers. Their study also found fake papers referencing predatory journals. Another study found questionable references to be a key feature for fake paper identification too (Dadkhah et al. 2023). However, their criteria for questionable references were an unfitting context of a reference and repeating authors in a reference list. Of all the features examined in our study, the relative differences between the fake paper group and the reference group are largest for this feature. Nevertheless, this feature should not be used uncritically for fake paper identification. First, it is probably more suitable for retrospective fake paper identification, since the reference lists become only conspicuous with the retraction of other fake papers. We did not examine when the referenced literature got retracted (before or after the submission of the paper referencing this literature). Second, even honest authors can accidentally reference fake papers before they are retracted (this happened rather rarely in our reference group). Third, there could also be honest retractions among the references, but we did not differentiate this and considered all retracted references as dubious. However, it should be noted that there was also one paper in the reference group that stood out as an outlier, as it also referred to five retracted publications (Liu et al. 2020). This article may be worth a closer look.

### Performance metrics

Both fake papers and reference papers performed very similarly in terms of accesses and citations. To make older and more recent publications comparable, we have calculated the average number of accesses and citations per year. For publications that had not been published for a full year at the time of data collection, these values were extrapolated. It is alarming how often the fake papers were accessed and cited per year. However, it is not clear if all citations were from honest authors. There are reports of citation cartels artificially pushing the number of citations of authors by referencing each other (Fister et al. 2016). Fake paper authors publish fake papers to artificially increase their number of publications. Therefore, it is also imaginable that they artificially increase the impact of these publications.

### Citations before and after the retractions of the fake papers

It took between 1 and 6 years to retract the fake papers. Unfortunately, that was enough time for them to be cited and contaminate the scientific record. Even worse, the fake papers continued to be cited even after they had been retracted. This has also been observed before (Candal-Pedreira et al. 2020) and shows that the damage caused by fake papers is permanent and cannot be fully contained even by their retraction.

### Did the fake papers damage the reputation of Chinese papers in NSAP?

We wondered whether the editors of NSAP would now treat Chinese papers unfavorably in response to the attacks by paper mills, which were mainly from China. We could not find any evidence of this in the two parameters we analyzed. After the journal became aware of the fake papers from China in 2020, the number of Chinese publications did not develop differently from the number of publications from other countries with many publications in the NSAP. Also, the time required to publish an article in NSAP has not increased for Chinese authors (it even decreased), which indicates that no worse prioritization has taken place. Among NSAP readers, the fake papers have not reduced the popularity of Chinese publications. At least the average citations per paper and per year of Chinese publications in NSAP have not changed worse than for publications from other countries.

### Limitations

All metadata we analyzed in this study were from a single journal. Therefore, the results may be quite specific to NSAP. Furthermore, the number of fake papers in our dataset was small. In addition, it is possible that our reference group also contained fake papers that have not yet been exposed. These could confuse the results. Further limitations affecting certain metadata have already been discussed in the respective sections.

Initially, we collected much more metadata to find a lot more characteristics than described in this study. For example, we investigated citation cartels, self-citations, retractions in the author records, and much more. However, the basis for determining all this data was reliable tracking of the authors' publications. Among others, Semantic Scholar offers an overview of all publications of an author. Unfortunately, it turned out that it did not track the authors’ publications reliably. In the Semantic Scholar database, we found authors with more than 5000 publications. In other cases, current NSAP authors have also been attributed with publications from 1875. This was obviously not plausible, and therefore, we excluded all metadata based on author records and did not evaluate them.

## Conclusion

For many of the characteristics we examined, the fake papers were inconspicuous and did not differ much from the papers in the reference group. That the fake papers in our dataset were able to pass peer review and get published shows that they were well fabricated. It is therefore not surprising that they were as inconspicuous at the metadata level as they were at first glance in terms of content. However, there were also characteristics in which the fake papers differed more from the reference papers. The features “countries,” “non-institutional email domains,” and “references to retracted papers” may be useful for detecting fake papers, but not as sole criteria. Even in combination, these three features would still be far too unspecific. With 2.9 million scientific articles published in 2020 alone (White 2021), it is important to reduce the false positive hits so that a realistic number of suspect papers can be examined in detail. Furthermore, features such as the origin of authors should only be used very carefully to detect fake papers, as this could discriminate against honest authors from certain countries. This also applies to features with geographical differences.

We hope for more research on fake papers. Especially further ways to detect these papers are required. Since fake papers cause lasting damage, it is particularly important to detect them before they are published. Automatic fake paper detection tools are already being developed for this purpose. Unfortunately, it is not transparent which features these tools use to detect fake papers (Else 2022). On the one hand, this is understandable, as one does not want to give the paper mills the opportunity to adapt to these tools. On the other hand, the scientific community cannot verify the features used. We have shown examples where discrimination could occur.

Recently, Pathmendra et al. (2023) reported that not only journals with a low impact factor, but also those with a high impact factor are affected by paper mill fraud.

## Take-home messages

- Fake papers have an average publication time. This suggests that they are inconspicuous to both reviewers and editors.
- In most of the metadata we examined, the fake papers did not differ relevantly from the supposedly honest papers.
- The features on which the two groups differed are only of limited use for identifying fake papers. For one thing, these features are far too unspecific, with too many false positives. For another, some of these features are discriminatory against honest authors from certain countries.
- Fake papers are still cited even after their retraction. Detecting them before they can be published is therefore all the more important.
- If a paper mill attacks a journal, it may submit several fake papers within a relatively short period of time. Therefore, if one fake paper is discovered in a journal, it may be worth looking more closely at other papers in the same journal that were submitted around the same time.

### Supplementary Information

Below is the link to the electronic supplementary material.

Supplementary file1 (XLSX 121 KB)

## Data Availability

All original data of this study are attached as a supplementary file.

## Abbreviations

NSAP: Naunyn-Schmiedeberg’s Archives of Pharmacology
CI: Confidence Interval
